# Radiation Dose to Critical Cardiac Structures from Three-Dimensional Conformal Radiation Therapy (3D-CRT), Intensity-Modulated Radiation Therapy (IMRT) and Volumetric Modulated Arc Therapy (VMAT) Techniques for Left-Sided Breast Cancer

**DOI:** 10.3390/jpm14010063

**Published:** 2024-01-03

**Authors:** Evgenia Konstantinou, Antonis Varveris, Georgia Solomou, Chrysostomos Antoniadis, Maria Tolia, Michalis Mazonakis

**Affiliations:** 1 Department of Medical Physics, Faculty of Medicine, University of Crete, 71003 Heraklion, Greece; 2 Department of Radiotherapy and Oncology, University General Hospital of Heraklion, 71110 Heraklion, Greece; 3 Department of Medical Physics, University General Hospital of Heraklion, 71110 Heraklion, Greece

**Keywords:** left breast cancer, LAD, LV, 3D-CRT, IMRT, VMAT

## Abstract

A comparison of the radiation exposure to the left anterior descending artery (LAD) and left ventricle (LV) was performed for twenty-three left breast cancer patients. For each participant, two tangential fields 3D-CRT, two- and seven-field IMRT and two and four partial arcs VMAT plans were created. Dose constraints for CTV, ipsilateral lung and heart were followed. The V_40Gy_, V_30Gy_, D_av_ of LAD and V_23Gy_, V_5Gy_, D_av_ of LV were calculated and extracted from the plans. Parametric and non-parametric tests were applied to compare the parameters derived from the five treatment techniques. All generated plans fulfilled the dose constraints. The D_av_ ranges of the LAD and LV from all examined techniques were 11.77–14.73 Gy and 5.37–6.40 Gy, respectively. The V_40Gy_ and V_30Gy_ ranges of the LAD were 2.90–12.91% and 10.80–18.51%, respectively. The V_23Gy_ and V_5Gy_ of the LV were 4.29–7.43% and 18.24–30.05%, respectively. The VMAT plans and seven-field IMRT significantly reduced the V_40Gy_, V_30Gy_ of LAD and V_23Gy_ of LV compared with the two-field treatments (*p* < 0.05). However, 3D-CRT plans provided statistically lower values for V_5Gy_ of LV over the other techniques (*p* < 0.05). The presented results provide a detailed dataset of the radiation burden of two critical cardiac structures from five radiotherapy techniques.

## 1. Introduction

Female breast cancer is the most frequently diagnosed cancer and the fifth leading cause of cancer death worldwide [1]. According to global statistics in 2020 [1], over 2.2 million breast cancer incidents were recorded, accounting for approximately 700 thousand deaths. An improved 5-year survival rate of 90.8% has been reported for females diagnosed with breast malignancies between 2013 and 2018 in USA [2]. This has been attributed to early cancer detection through mammographic screening and to advances in applied anticancer treatment [3,4].

Whole breast radiation therapy (WBRT) after breast-conserving surgery (BCS) is indicated for early-stage breast cancer patients [5]. The adjuvant radiotherapy enhances the reduction of local recurrence as well as the risk of breast cancer death [6,7]. However, radiotherapy may induce cardiac toxicity [8,9,10]. Darby et al. [8] found that the relative risk for developing major coronary events in females irradiated for breast cancer is 7.4% per Gy received by the whole heart without apparent threshold for breast cancer patients who received radiotherapy. After the exposure, the risk started rising within the first five years and continued for more than 20 years. Left-sided breast cancer patients are at an increased risk of developing heart diseases compared with right-sided patients [9,10,11]. Moreover, higher rates of cardiac death were observed for these patients [9,12,13]. Bouillon et al. [13] showed that patients who were irradiated for left breast cancer had a 1.56-fold greater risk of cardiac disease death than patients treated for right breast malignancies.

Radiation-induced cardiotoxicity is associated with the exposure of the heart and its substructures, mainly the left anterior descending artery (LAD) and the left ventricle (LV). The LAD artery, which is located at the anterior part of the heart, receives significant radiation dose due to radiotherapy [14,15,16]. Irradiating the LAD artery can cause coronary artery disease which then results in subsequent ischemic disease [8,11,14]. Complications in the left ventricle related to radiotherapy such as perfusion defects and segmental alterations have previously been recorded [17,18,19]. Subclinical LV dysfunction appeared 6 months after the radiotherapy, as reported by Walker et al. [18]. Recent studies [20,21] have proved that the deep inspiration breath-hold technique effectively contributes to a dose-sparing effect on the heart and these two cardiac substructures compared with free breath. However, this technique is not widely applicable at present.

Breast cancer irradiation may be based on the conventional three-dimensional conformal radiotherapy (3D-CRT) [22]. More advanced techniques such as intensity-modulated radiation therapy (IMRT) and volumetric modulated arc therapy (VMAT) may also be applied for the management of this malignancy [22]. Limited information exists in the literature about the comparison of the above three treatment techniques in respect to the radiation exposure of LAD and LV. Kuzba-Kryszak et al. [23] provided data about the dose to LAD from 3D-CRT, IMRT and VMAT. Their investigation was carried out for the deep inspiration breath-hold technique. No information was given about the LV. To our knowledge, no attempt has been conducted to directly compare the three radiotherapy techniques in free breathing conditions.

The objective of this study was to compare the dosimetric parameters of the LAD and LV from 3D-CRT, IMRT with two and seven fields and VMAT with two and four partial arcs for left breast cancer patients.

## 2. Materials and Methods

### 2.1. Patients

Twenty-three left-sided breast cancer patients who had previously undergone radiotherapy at the Radiation Oncology department of the University General Hospital of Heraklion were included in this study. The treatment of all patients had been completed prior to the beginning of this work. Whole breast irradiation was performed. For the implementation of this study, approval was granted by the Ethics Committee at the University General Hospital of Heraklion. All study participants were diagnosed with early stage left breast cancer (T1–T2). The patients’ tumor grade was 1–3. These patients had undergone breast-conserving surgery followed by adjuvant whole breast irradiation. Patients with metastatic disease were excluded. Moreover, patients needing axillary or lymph node irradiation were not included in the patient group of this work. The patients’ mean age was 55 ± 12 years old (range: 31–83); 61% of the total number of patients were under the mean patient age of 55 years old.

### 2.2. Planning Computed Tomorgaphy

**Figure 1:**
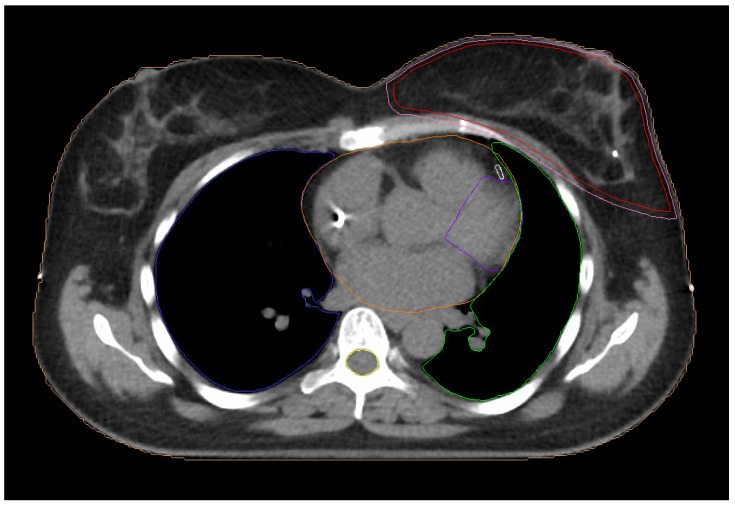
Computed tomography image with contoured structures: CTV (red); PTV (pink); heart (orange); ipsilateral lung (green); contralateral lung (blue); LAD (white); left ventricle (purple); spinal cord (yellow).

In supine position, patients were immobilized with both arms upward on a breast board and the head facing to the right. Planning images were acquired using a 16-slice computed tomography unit (Somaton Sensation 16, Siemens, Forcheim, Germany). The clinical target volume (CTV) ranged between 237 cm^3^ and 2535 cm^3^, with a mean value of 918 ± 506 cm^3^. The planning target volume (PTV) was generated by adding a 3 mm margin around the CTV. The heart and the ipsilateral lung were determined as the organs at risk. The process of contouring on the computed tomography images was based on previously published guidelines of the European Society for Therapeutic Radiology and Oncology [24]. The contouring of the target volume and all the involved surrounding organs were carried out with the aid of the treatment planning system (Monaco 5.11, Elekta AB, Stockholm, Sweden). Manual delineation on computed tomography scans was performed by a radiation oncologist experienced in breast cancer treatment. In addition, the same radiation oncologist contoured the two cardiac structures, the LAD and LV. A senior radiation oncologist consistently carried out a review of the contours of all structures on a slice-by-slice basis and performed the necessary changes and corrections. The LAD artery and the LV as well as their ramifications were manually delineated on a slice-by-slice basis in accordance with previously reported data [25,26]. Figure 1 shows all the contoured structures.

### 2.3. Treatment Planning

Treatment planning was made using the Monaco system for beam delivery on an Infinity linear accelerator (Elekta AB, Stockholm, Sweden). The treatment plans were generated with 6 MV photons which is the standard currently employed in our department for all breast cancer patients. The above photon energy is usually preferred for breast cancer management to avoid the underdose of superficial tissues beneath the skin surface [27]. The isocenter was placed in the center of the PTV. Radiotherapy treatment plans were made with five different techniques for each patient. The treatment techniques examined in this work have already been employed in previously published studies [23,28,29]. The radiation dose to the LV and LAD artery was not considered in the optimization process during treatment planning.

The prescription dose for CTV was 50 Gy given in 25 fractions of 2 Gy. The dose constraints for the target volume and the critical organs were based on the Danish Breast Cancer Group Hypo trial [30] for whole breast radiation therapy. In this model, 95% of the CTV had to received 95% of the total dose (D_95%_ ≥ 47.5 Gy), while the maximum dose should not be greater than 55 Gy. The volume of ipsilateral lung receiving a dose of 20 Gy had to be kept less than 20% (V_20Gy_ < 20%). The heart’s constraints were V_20Gy_ < 10% and V_40Gy_ < 5%. Additionally, through the treatment planning system we recorded other dosimetric parameters which are shown in Table 1. The LAD and LV parameters were based on the recommendations of Piroth et al. [31] as representative for heart protection from high therapeutic doses. They defined the dosimetric parameters of both cardiac structures on the basis of the clinical evidence on cardiac toxicities owing to radiation [31]. The same dosimetric parameters for the LAD and LV structures have previously been used in studies dealing with radiotherapy for breast carcinomas [32,33,34].

#### 2.3.1. 3D-CRT Technique

**Figure 2:**
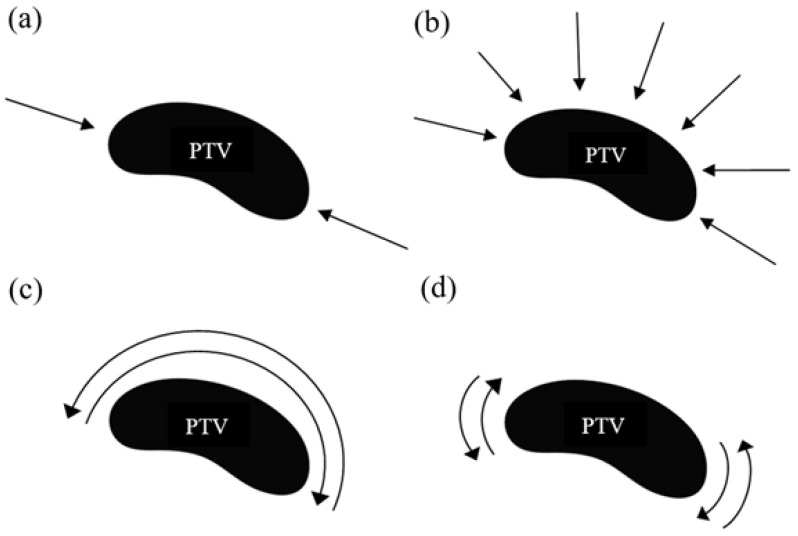
Beam and arc arrangements for the planning techniques: (**a**) 3D-CRT and two-fields IMRT (2F-IMRT); (**b**) seven-fields IMRT (7F-IMRT); (**c**) two partial arcs VMAT (VMAT1); (**d**) four partial arcs VMAT (VMAT2).

All plans with 3D conformal radiation therapy consisted of two tangential fields without wedges (Figure 2a). Gantry’s angle for the internal field ranged from 300° to 315°, while for the external field it was between 123° and 140°. The beams’ angles were selected on the basis of a beam-eye-view approach. The directions and weights of the applied beams were chosen to optimize the coverage of CTV and to restrict the radiation dose received by the surrounding organs. Furthermore, an expansion was added in both PTV and anteriorly from the chest wall to create the “flash” region. To avoid hot spots and creating homogeneous dose distribution we generated low weight segments using multileaf collimator. The number of segments varied among patients. No more than four segments were added per treatment field. Plans were designed by a forward planning system using a collapsed cone algorithm.

#### 2.3.2. Two-Field IMRT Technique (2F-IMRT)

The plans for this technique were implemented with two opposed fields (Figure 2a). For each individual, the field angles were the same as the 3D-CRT technique. In this context, in seven patients an additional field was added to improve higher coverage in CTV with an angle of 340° or 345°. Inverse planning was performed with dynamic multileaf collimator delivery. The maximum number of control points was 20 per beam. Dose calculation was carried out with a Monte Carlo algorithm and then it was optimized with the fluence optimizer algorithm using a 0.5 cm grid. The collimator was angled to adjust the chest wall shape.

#### 2.3.3. Seven-Field IMRT Technique (7F-IMRT)

Multibeam IMRT comprised seven coplanar beams. The beams were equally spaced through a sector angle of 190° around the target volume [5]. For each beam, the control points were up to 20. A typical beam arrangement is illustrated in Figure 2b.

#### 2.3.4. Two Partial-Arc VMAT Technique (VMAT1)

A VMAT plan consisting of a dual partial arc was applied for all participants. The length of each arc was always 200°. The starting angle ranged between 293° and 306°. The two coplanar arcs rotated in clockwise and counterclockwise directions. This dual arc approach is widely applied for VMAT planning [35,36,37]. Figure 2c presents the positioning of partial arcs. VMAT plans were created by using a 0.5 cm grid and the maximum number of control points was 150. For all plans, the collimator was in 0°.

#### 2.3.5. Four Partial-Arc VMAT Technique (VMAT2)

Two double partial arcs of 50° each were used in VMAT plans (Figure 2d). Each double arc comprised of a counterclockwise and a clockwise arc. For the internal arc, the irradiation starting point varied from 283° to 295°, while the external arc was 103° to 120°. All treatment plans were designed inversely using a 0.5 cm grid. The maximum number of control points was 100 for each arc and the collimator was in 0°. The gantry rotated both clockwise and anticlockwise. The dual arc approach is currently employed in our department for the creation of all VMAT plans [5].

### 2.4. Statistical Analysis

Statistical analysis was performed using the Statistical Package for the Social Sciences (SPSS) for Windows version 28.0 (IMB Corp., Armonk, NY, USA). The mean and standard deviation were calculated for all the dosimetric parameters of all contoured structures. Further analysis was conducted for the LAD and LV. The differences in the V_40Gy_, V_30Gy_ and D_av_ of the LAD as calculated for the five treatment planning strategies were evaluated. The above process was carried out for the parameters related to LV. Statistical tests have been successfully used to indicate significant differences between dosimtetric and/or radiobiological parameters determined by different radiation therapy techniques [38]. Normalization analysis was carried out with the Shapiro–Wilk test for all the variables. Subsequently, the paired sample *t*-test and the Wilcoxon signed ranks test were used for variables comparison of treatment plans. A significance level of 5% was determined.

## 3. Results

### 3.1. Treatment Evaluation

**Figure 3:**
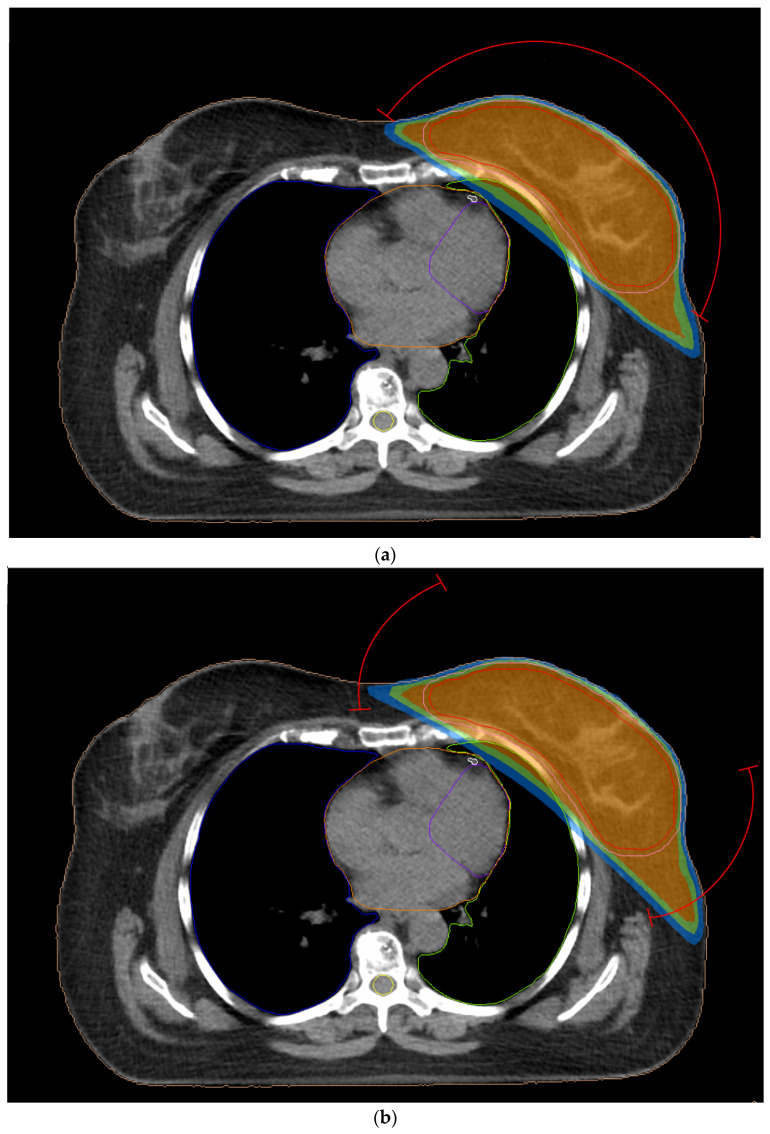
Computed tomography image with contoured structures: CTV (red); PTV (pink); heart (orange); ipsilateral lung (green); contralateral lung (blue); LAD (white); left ventricle (purple); spinal cord (yellow). Treatment plans of left breast cancer with dose distribution around the target volume (red) which treated with (**a**) two partial-arc VMAT and (**b**) four partial-arc VMAT. The isodoses of orange, green and blue color correspond to 95%, 85% and 70% of total dose, respectively.

All treatment plans fulfilled the dose constraints, and they were considered clinically acceptable. VMAT1 and VMAT2 plans are presented in Figure 3 with isodoses covering the target volume. Table 2 summarizes the mean values of the planning parameters for the CTV, PTV and the surrounding organs. The 7F-IMRT, VMAT2, VMAT1 techniques led to mean D_95%_ of 99.12%, 99.00%, 98.86%, respectively. The 2F-IMRT and 3D-CRT plans gave lower values of 97.04% and 96.56% accordingly. Similar results were found for the D_95%_ of the PTV. The highest dose coverage for PTV of 96.45% was achieved for VMAT2 followed by 7F-IMRT and VMAT1 with mean values of more than 95.46%. The mean V_40Gy_ of the heart associated with the two VMAT techniques and 7F-IMRT varied from 0.52 to 0.67% whereas the range for V_20Gy_ was 3.17 to 3.38%. The V_40Gy_ and V_20Gy_ from techniques comprising only two tangential treatment fields were at least 1.65% and 4.65%. The D_av_ of the heart from the conventional 3D-CRT was 3.70 Gy. The IMRT and VMAT techniques led to D_av_ of 5.15 Gy or more. The 7F-IMRT, VMAT1 and VMAT2 plans provided a mean V_20Gy_ of the ipsilateral lung of 8.90%, 9.65% and 10.00%, respectively. The corresponding value from both 3D-CRT and 2F-IMRT was more than 11.22%.

### 3.2. Exposure of Cardiac Structures

The calculated mean value and standard deviation of LAD and LV dosimetric parameters are summarized in Table 3. For both critical structures, the differences between the parameters derived from the five examined techniques are presented in Table 4.

#### 3.2.1. Left Anterior Descending Artery (LAD)

The 7F-IMRT and two VMAT techniques led to mean V_40Gy_ and V_30Gy_ values of 2.90–4.98% and 10.80–12.84%, respectively. For the techniques that consisted of two fields, the V_40Gy_ and V_30Gy_ were no less than 10.30% and 17.48%. Significant statistical differences were observed between the LAD parameters obtained by IMRT, VMAT1 and VMAT2 plans from these calculated by two-field treatments (Table 4). In addition, VMAT1 plans provided a statistically lower value for V_40Gy_ compared with 7F-IMRT plans (2.90% vs. 4.98%, *p* = 0.039). The range of mean D_av_ was 11.77–14.73 Gy, using the five irradiation approaches. Statistical difference was found by comparing VMAT2 plans with 3D-CRT, 2F-IMRT and 7F-IMRT. This was also found between VMAT1 and 2F-IMRT and 7F-IMRT (*p* = 0.039; 0.014 respectively).

#### 3.2.2. Left Ventricle (LV)

Assessing the mean V_23Gy_ of the LV, the lowest mean values were derived from VMAT2, VMAT1 and 7F-IMRT plans at 4.29%, 4.47% and 4.53%, respectively. The 2F-IMRT and 3D-CRT plans gave mean values of 7.26% and 7.43%. According to Table 4, significant difference was observed for 7F-IMRT, VMAT1 and VMAT2 plans (*p* < 0.001) over the 3D-CRT and 2F-IMRT. The mean V_5Gy_ was 18.24% for conformal treatment and was increased from 25.80 to 30.05% with the use of other techniques. Statistical difference was exhibited when comparing 3D-CRT with the other four techniques (Table 4). Among the five examined techniques, the D_av_ varied between 5.37 Gy and 6.40 Gy. There was a significant difference between VMAT2 plans with 2F-IMRT, 7F-IMRT and VMAT1 plans (*p* = 0.002) and 3D-CRT with 2F-IMRT (*p* = 0.007).

## 4. Discussion

The high doses received by the LAD and LV structures after radiotherapy for left breast cancer may increase the risk of heart complications despite the low mean heart dose. This study examined the radiation exposure of both LAD and LV from radiotherapy for left breast cancer patients. Five different treatment techniques were applied including the 3D-CRT, IMRT with two and seven fields and VMAT with two and four partial arcs. For all the generated plans, we did not apply any optimization objective for the LAD and LV structures.

The treatment plans of all study participants satisfied the dose constraints, and they were considered as acceptable for clinical use. For seven patients, a third field was added in the 2F-IMRT plans to increase the dose coverage in the CTV. The two VMAT techniques and 7F-IMRT provided superior target volume coverage as well as leading to lower mean values for V_20Gy_ of left lung and V_40Gy_, V_20Gy_ of heart. In addition, 7F-IMRT, VMAT1 and VMAT2 plans significantly contributed to the LAD artery dose sparing when evaluating the V_40Gy_ and V_30Gy_. A superiority of VMAT1 plans instead of 7F-IMRT in respect to V_40Gy_ was found. For the V_23Gy_ of the LV, a statistically significant dose reduction was observed with the use of the 7F-IMRT and VMAT planning methods over the two tangential techniques. On the other hand, the 3D-CRT technique resulted in significantly lower V_5Gy_ as opposed to other techniques. Furthermore, it is noteworthy to mention that the D_av_ received by the LAD was substantially higher than the D_av_ of the whole heart (Table 2 and Table 3). Similar results were found, though with less variation, for the LV.

Kuzba-Kryszak et al. [23] compared the 3D-CRT, IMRT and VMAT planning techniques in deep inspiration breath-hold conditions. They reported a D_av_ for the LAD of 19.72 Gy, 13.00 Gy and 19.13 Gy for the above techniques, respectively. A slight dose reduction in the LAD artery was observed by applying inverse planning techniques. However, direct comparison with our study cannot be made because their investigation was carried out in patients receiving breath-hold radiotherapy to 42.5 Gy with 6 and 15 MV beams. Another study was conducted by Zhang et al. [39]. They estimated LV exposure with the use of 3D-CRT and two IMRT techniques. All IMRT plans included six beams, while the second IMRT used a lower number of segments with greater minimum area. Our results related to the D_av_ of the LV from 3D-CRT and 7F-IMRT plans were lower than their results. The D_av_ of the LV in their study was 12.54 Gy, 15.56 Gy and 16.37 Gy for two tangential fields 3D-CRT and the two IMRT plans respectively. In addition, their 3D-CRT plans provided significantly lower D_av_ and V_5Gy_ compared with the IMRT plans. However, no significant difference was found between the two IMRT plans and 3D-CRT for the high-dose volume parameter (V_25Gy_). Regarding our results, statistical difference between 7F-IMRT and 3D-CRT was observed for V_23Gy_ and V_5Gy_. Moreover, Karpf et al. [40] showed that multibeam step-and-shoot IMRT plans significantly reduced the D_av_ of the LAD when compared with four semi-arc VMAT plans (5.80 Gy vs. 7.32 Gy, *p* = 0.03). In our study, the 7F-IMRT led to a significantly higher D_av_ of the LAD compared with the VMAT plans.

Regarding our findings, the examined dosimetric parameters of the two cardiac structures were higher than the values recommended by Piroth et al. [31]. This was not observed for V_23Gy_ of the LV for the 7F-IMRT, VMAT1 and VMAT2 plans. The planning results of this study were extracted without considering the radiation dose for the LAD and LV in the optimalization process. This indicates that the treatment plans should be evaluated and for the heart substructures beyond the whole heart.

The current study may be limited by the relatively small size of left-sided breast cancer patients. All participants received whole breast irradiation. Patients needing lymph node treatment were not included. Additionally, no consideration was taken of breast size. A subsequent study could examine the effect of breast size on the radiation dose to LAD and LV for each of the five radiotherapy techniques used in this work. The female patients could be divided in different categories on the basis of the target volume. Further research is required to evaluate whether previously reported cardioprotective strategies [41] related to prone positioning, gating and proton therapy may have a considerable impact on LAD and LV exposure. It should also be mentioned that a follow-up study of patients irradiated for left-sided breast cancer, including the appropriate heart examinations, could reveal the association of radiation-induced cardiac complications with the dose absorbed by the LAD or LV.

## 5. Conclusions

A total of 115 radiotherapy plans were generated for left-sided breast cancer patients using 3D-CRT, 2F-IMRT, 7F-IMRT, VMAT1 and VMAT2. All the treatment plans were deemed clinically acceptable. Comparing the five irradiation techniques, the 7F-IMRT, VMAT1 and VMAT2 techniques proved to significantly reduce the V_40Gy_ and V_30Gy_ of the LAD and the V_23Gy_ of the LV. In contrast, the use of 3D-CRT was significantly superior in low-dose volume (V_5Gy_) for LV compared with other techniques. In conclusion, this research provides information for the LAD and LV radiation burden regarding the different treatment techniques with different beams and arc arrangements.

## Figures and Tables

**Table 1:** Dosimetric parameters of planning target volume (PTV), heart and cardiac structures.

Structure	Dosimetric Parameters
PTV	D_95%_
Heart	D_av_
LAD	V_40Gy_
	V_30Gy_
	D_av_
LV	V_23Gy_
	V_5Gy_
	D_av_

D_95%_: target percentage receiving 95% of total dose; D_av_: average dose; V_iGy_: organ volume receiving less than iGy.

**Table 2:** Dosimetric parameters for CTV, PTV, whole heart and ipsilateral lung as derived from five different treatment planning techniques. Dose values are given as mean ± one standard deviation.

Structure	Parameter	Mean ± Standard Deviation				
		3D-CRT	2F-IMRT	7F-IMRT	VMAT1	VMAT2
CTV	D_95%_ (%)	96.56 ± 1.45	97.04 ± 1.73	99.12 ± 1.00	98.86 ± 1.30	99.00 ± 1.13
PTV	D_95%_ (%)	92.11 ± 1.95	92.68 ± 1.62	96.04 ± 0.98	95.46 ± 1.09	96.45 ± 1.30
Heart	V_40Gy_ (%)	1.88 ± 1.31	1.65 ± 1.34	0.67 ± 0.68	0.55 ± 0.56	0.52 ± 0.58
	V_20Gy_ (%)	4.65 ± 2.19	4.67 ± 2.37	3.38 ± 1.72	3.28 ± 1.94	3.17 ± 1.82
	D_av_ (Gy)	3.70 ± 0.98	6.43 ± 10.60	5.21 ± 1.04	5.15 ± 1.08	5.99 ± 9.54
Isp. lung	V_20Gy_ (%)	11.40 ± 4.76	11.22 ± 4.51	8.90 ± 3.26	9.65 ± 3.36	10.00 ± 3.47

CTV: clinical target volume; PTV: planning target volume; 3D-CRT: three-dimensional conformal radiotherapy; 2F: two fields; 7F: seven fields; IMRT: intensity-modulated radiation therapy; VMAT: volumetric modulated arc therapy; VMAT1: VMAT with two partial arcs; VMAT2: VMAT with four partial arcs.

**Table 3:** Dosimetric parameters for LAD and LV as derived from five different treatment planning techniques. Dose values are given as mean ± one standard deviation.

Structure	Parameter	Mean ± Standard Deviation				
		3D-CRT	2F-IMRT	7F-IMRT	VMAT1	VMAT2
LAD	V_40Gy_ (%)	12.91 ± 11.76	10.30 ± 10.74	4.98 ± 8.04	2.90 ± 5.91	3.10 ± 6.08
	V_30Gy_ (%)	18.51 ± 13.00	17.48 ± 13.73	12.84 ± 11.96	10.80 ± 10.84	10.88 ± 12.07
	D_av_ (Gy)	12.94 ± 5.33	14.73 ± 9.40	12.92 ± 5.51	12.02 ± 4.91	11.77 ± 5.05
LV	V_23Gy_ (%)	7.43 ± 4.18	7.26 ± 4.50	4.53 ± 3.27	4.47 ± 3.36	4.29 ± 3.40
	V_5Gy_ (%)	18.24 ± 6.26	28.77 ± 17.46	30.05 ± 14.38	29.99 ± 14.75	25.80 ± 14.05
	D_av_ (Gy)	5.69 ± 1.92	6.40 ± 1.75	5.93 ± 1.77	5.89 ± 1.80	5.37 ± 1.76

LAD: left anterior descending; LV: left ventricle; 3D-CRT: three-dimensional conformal radiotherapy; 2F: two fields; 7F: seven fields; IMRT: intensity-modulated radiation therapy; VMAT: volumetric modulated arc therapy; VMAT1: VMAT with two partial arcs; VMAT2: VMAT with four partial arcs.

**Table 4:** Statistical comparison between the five different treatment planning techniques for the dosimetric parameters of the LAD and LV.

Techniques Comparison	*p*-Value					
	LAD			LV		
	V_40Gy_	V_30Gy_	D_av_	V_23Gy_	V_5Gy_	D_av_
3D-CRT vs. 2F-IMRT	0.007 *	0.101	0.316	0.546	<0.001 *	0.007 *
3D-CRT vs. 7F-IMRT	<0.001 *	<0.001 *	0.967	<0.001 *	<0.001 *	0.277
3D-CRT vs. VMAT1	<0.001 *	<0.001 *	0.125	<0.001 *	<0.001 *	0.412
3D-CRT vs. VMAT2	<0.001 *	<0.001 *	0.016 *	<0.001 *	0.002 *	0.110
2F-IMRT vs. 7F-IMRT	<0.001 *	<0.001 *	0.484	<0.001 *	0.162	0.144
2F-IMRT vs. VMAT1	<0.001 *	<0.001 *	0.039 *	<0.001 *	0.144	0.124
2F-IMRT vs. VMAT2	<0.001 *	<0.001 *	0.008 *	<0.001 *	0.533	0.002 *
7F-IMRT vs. VMAT1	0.039 *	0.071	0.014 *	0.436	0.966	0.809
7F-IMRT vs. VMAT2	0.019 *	0.198	0.008 *	0.263	0.104	0.002 *
VMAT1 vs. VMAT2	0.767	0.629	0.545	0.487	0.098	0.002 *

LAD: left anterior descending; LV: left ventricle; 3D-CRT: three-dimensional conformal radiotherapy; 2F: two fields; 7F: seven fields; IMRT: intensity-modulated radiation therapy; VMAT: volumetric modulated arc therapy; VMAT1: VMAT with two partial arcs; VMAT2: VMAT with four partial arcs. * Statistically significant differences (*p* < 0.05).

## Data Availability

Data of this study are available from the corresponding author upon request.
